# Comparison of Thoracic Epidural and Bilateral Erector Spinae Plane Block for Postoperative Analgesia in Laparoscopic Surgeries: A Randomized Double-Blind Study

**DOI:** 10.7759/cureus.114003

**Published:** 2026-08-05

**Authors:** Prasanna Vadhanan, Rahul Kumar, Mannar Mannan, Keerthana Kodur

**Affiliations:** 1 Department of Anesthesiology, Vinayaka Missions Medical College and Hospital, Karaikal, IND

**Keywords:** erector spinae plane block, laparoscopic surgery, postoperative pain, regional anaesthesia, thoracic epidural analgesia

## Abstract

Background

Effective postoperative pain control is essential for enhanced recovery after laparoscopic abdominal surgeries. Thoracic epidural analgesia (TEA) has traditionally been considered a gold-standard technique for perioperative analgesia in abdominal procedures because of its ability to provide excellent pain relief. However, its use may be limited by technical difficulty, risk of hypotension, and other hemodynamic concerns. In recent years, the ultrasound-guided erector spinae plane (ESP) block has gained attention as a simpler and potentially safer regional analgesic technique. The study aimed to compare the efficacy and safety of single-dose TEA and bilateral ESP block for postoperative analgesia in adult patients undergoing laparoscopic abdominal surgery.

Methods

This randomized clinical study included 60 patients aged 15-80 years who were scheduled for laparoscopic abdominal surgery. Participants were equally assigned to two groups: Group A receiving single-shot TEA, and Group B receiving bilateral ultrasound-guided ESP block at the thoracic level. Standardized anesthetic management was followed in both groups, including uniform intraoperative opioid administration, neuromuscular blockade, and hemodynamic control. The primary outcome was comparison of postoperative pain scores between the two groups over the first 24 hours using the visual analogue scale (VAS). Secondary outcomes included time to first rescue analgesia, patient comfort during block administration, and procedure-related complications such as backache and hypotension.

Results

Postoperative analgesia was better in the ESP block at six and 12 hours after surgery (p<0.001). However, it was better with epidural analgesia immediately following surgery (p<0.05). There was no significant difference in VAS scores at 24 hours between the groups (p>0.05). The time to first rescue analgesia was significantly longer with ESP block (nine hours vs five hours; p<0.001). Patient comfort during block administration was better with the ESP block. The ESP block had significantly fewer postoperative complications (post-procedural backache and hypotension) than epidural analgesia (p<0.001).

Conclusion

Bilateral ESP block provided more sustained postoperative analgesia than single-dose TEA in laparoscopic abdominal surgery. Although thoracic epidural offered better immediate pain relief, the ESP block resulted in longer analgesia, better comfort, and fewer complications, making it a safer alternative.

## Introduction

Effective postoperative pain control is essential in abdominal surgery, as adequate analgesia promotes early recovery, improves patient comfort, and reduces opioid requirement [1]. Regional anesthesia techniques are an important part of multimodal analgesia for abdominal surgeries, and thoracic epidural analgesia (TEA) has traditionally been regarded as a standard technique because of its ability to provide both somatic and visceral pain relief [2].

However, TEA is associated with several limitations, including hypotension, urinary retention, and technical difficulty in patients with challenging thoracic anatomy or when neuraxial blockade is undesirable [3]. These concerns have encouraged the search for safer and simpler alternatives.

The erector spinae plane (ESP) block has recently gained attention as a promising regional technique for thoracic and abdominal procedures [4]. By depositing local anesthetic deep into the erector spinae muscle, it can provide effective postoperative analgesia through spread to the paravertebral and epidural spaces, thereby contributing to both somatic and visceral pain control [5]. Compared with TEA, the ESP block is technically simpler, less invasive, and associated with less hemodynamic disturbance, making it particularly attractive for laparoscopic surgeries [6].

Although TEA remains widely used for perioperative pain management, comparative evidence between TEA and ESP block in laparoscopic abdominal surgery remains limited and inconsistent [7]. As laparoscopic procedures require effective analgesia with minimal adverse effects, further evaluation of these techniques is warranted [8]. We hypothesized that the bilateral ESP block would provide postoperative analgesia comparable to single-shot TEA while offering a superior safety profile and improved patient comfort in patients undergoing laparoscopic abdominal surgery. Therefore, this study was undertaken to compare TEA and bilateral ESP for postoperative analgesia in patients undergoing laparoscopic abdominal surgery.

## Materials and methods

Study design and setting

A prospective, randomized, double-blind controlled trial was carried out in the Department of Anaesthesiology of a tertiary care teaching hospital from November 2024 to April 2025. Ethical clearance for the study was obtained from the Institutional Ethics Committee, and the trial was registered with the Clinical Trials Registry of India (CTRI/2024/11/076452). The study adhered to the principles of the Declaration of Helsinki, and written informed consent was obtained from all participants before enrolment.

Sample size

Sample size estimation using G*Power software showed that 60 patients, with 30 in each group, would provide adequate power to detect a clinically meaningful difference in postoperative VAS scores at a significance level of 0.05.

Study population

Patients of either sex aged 15-80 years undergoing laparoscopic surgery and belonging to the American Society of Anaesthesiologists (ASA) physical status I, II, or III were included [9]. Patients who refused consent, had a known allergy to the study medications, were classified as ASA physical status IV, had spinal abnormalities, or had contraindications to the study drug were excluded.

Randomization and blinding

Participants who met the eligibility criteria were randomly assigned in a 1:1 ratio to either Group A or Group B using a computer-generated block randomization sequence. The allocation sequence was prepared before patient enrollment, and allocation concealment was maintained using sequentially numbered, sealed, opaque envelopes. Each envelope was opened only by the anesthesiologist performing the assigned regional block immediately before the procedure.

Patients and postoperative outcome assessors remained blinded to group allocation throughout the study. To maintain patient blinding, local infiltration was performed at three sites in all participants (one midline and two bilateral paramedian sites), irrespective of the allocated intervention. The anesthesiologist performing the block was not involved in postoperative data collection or outcome assessment, which was conducted by independent investigators blinded to group allocation.

Regional techniques

A single-shot thoracic epidural technique was selected to provide a standardized comparison with the single-injection bilateral ESP block, allowing both interventions to be evaluated under comparable conditions without the influence of continuous catheter-based analgesia.

Patients in Group A received single-shot TEA. Under strict aseptic precautions, the epidural space was identified at the T7-T8 level using a midline approach and the loss of resistance technique. After negative aspiration, 10 mL of 0.25% bupivacaine was administered. No epidural catheter was inserted, as the study was designed to compare single-injection TEA with a single-injection bilateral ESP block.

Patients in Group B received bilateral ultrasound-guided ESP block. A high-frequency linear ultrasound probe was used to identify the T7 transverse process. After skin infiltration, a block needle was advanced in-plane until it contacted the transverse process deep to the erector spinae muscle. Correct placement was confirmed by hydro dissection, following which 20 mL of 0.25% bupivacaine was injected on each side, for a total volume of 40 mL.

Anesthetic management

Following successful administration of the assigned regional analgesic technique (TEA or bilateral ESP block), general anesthesia was standardized in both groups. Following routine monitoring and preoxygenation, anesthesia was induced with intravenous Propofol and Butorphanol according to institutional protocol. Neuromuscular blockade was achieved with vecuronium. Anesthesia was maintained with oxygen, nitrous oxide, and either isoflurane or sevoflurane titrated to maintain adequate anesthetic depth. No additional intraoperative opioids or analgesics were administered after induction. Intraoperative hemodynamic management was uniform in both groups, and all patients were monitored with noninvasive blood pressure, electrocardiography (ECG), pulse oximetry, and end-tidal carbon dioxide (ETCO₂) throughout the perioperative period.

Postoperative analgesia and outcome measures

Postoperative pain was assessed using a 0-10 visual analogue scale (VAS), where 0 indicated no pain, and 10 indicated the worst imaginable pain [10]. VAS scores were recorded at baseline, immediately after surgery (0 hour), and at six, 12 and 24 hours postoperatively.

The primary outcome was comparison of postoperative pain scores between the two groups over the first 24 hours. All patients received the same standardized postoperative analgesic protocol. Rescue analgesia was administered when the VAS score exceeded 5 or when the patient reported significant pain. Intravenous paracetamol 1 g was used as the first-line rescue analgesic, followed by intramuscular butorphanol (approximately 0.02 mg/kg) if further pain relief was required. Time to first rescue analgesia was recorded.

Secondary outcomes included patient comfort during block administration and the incidence of procedure-related complications such as backache and hypotension. All adverse events were documented.

Statistical analysis

The statistical analysis was carried out using IBM SPSS Statistics for Windows, Version 24 (Released 2016; IBM Corp., Armonk, New York, United States). Descriptive statistical analysis was carried out in this study. All data presented in the manuscript represent the original study observations. Continuous variables are reported as mean ± standard deviation (min-max) or median (25-75 quartiles), depending on data distribution, while categorical variables are presented as frequencies and percentages. A chi-square test was performed to compare the categorical variables among the two groups. An independent sample t test was used to compare continuous variables between the two groups. The distribution of continuous variables was assessed using the Shapiro-Wilk test before selecting the appropriate statistical tests. The Mann-Whitney U test was used to compare the differences between two independent groups for non-normal distributions. p-values < 0.05 were considered significant for all statistical tests.

## Results

**Figure 1:**
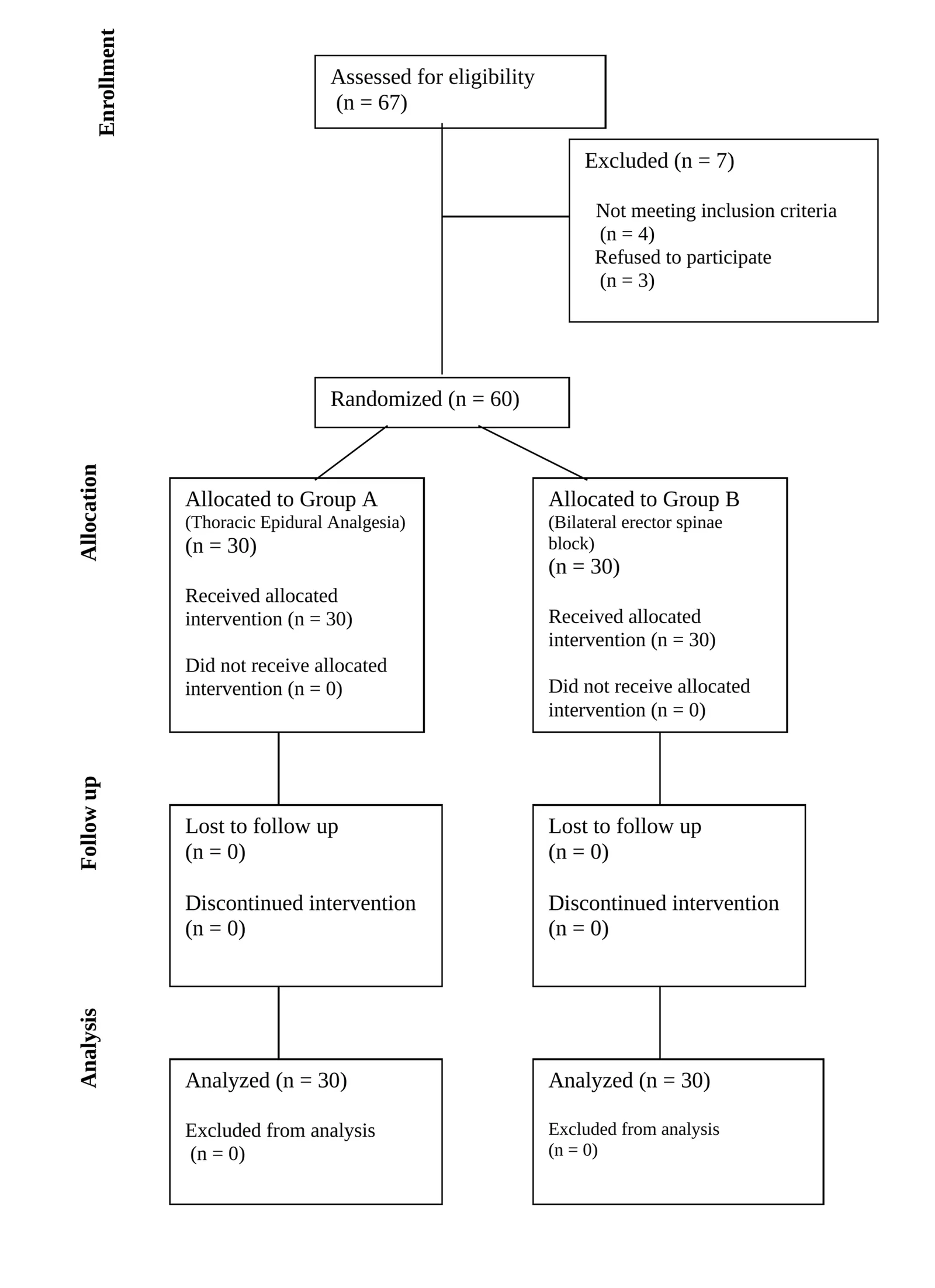
CONSORT flow diagram of the study CONSORT: Consolidated Standards of Reporting Trials

Of 67 patients assessed for eligibility, 60 were recruited and randomly allocated into two equal groups (n = 30) (Figure 1).

Baseline characteristics

A total of 60 (100%) patients completed the study. Baseline demographic characteristics and preoperative clinical status were comparable between the two groups. There were no statistically significant differences in age, sex distribution, ASA physical status, or the distribution of surgical procedures between the two groups, indicating that the study groups were well matched for comparative analysis (Table 1). No missing data were encountered, and all randomized patients completed the study and were included in the final analysis.

**Table 1:** Baseline demographic characteristics ^#^Independent samples t-test ^$^Chi-square test ESP: erector spinae block; ASA: American Society of Anesthesiologists; SD: standard deviation

Variable	Group A: Thoracic epidural analgesia (n = 30)	Group B: Bilateral ESP block (n = 30)	p-value
Age (years), mean ± SD	40.57 ± 12.65	39.10 ± 12.66	0.962^#^
Male, n (%)	8 (26.7)	11 (36.7)	0.405^$^
Female, n (%)	22 (73.3)	19 (63.3)	
ASA I, n (%)	1 (3.3)	4 (13.3)	0.369^$^
ASA II, n (%)	25 (83.3)	22 (73.3)	
ASA III, n (%)	4 (13.3)	4 (13.3)	

Baseline hemodynamic parameters

Preoperative baseline hemodynamic parameters recorded before induction of anesthesia were comparable between the two groups. There were no statistically significant differences in systolic blood pressure, diastolic blood pressure, heart rate, or oxygen saturation, confirming baseline physiologic comparability (Table 2).

**Table 2:** Baseline hemodynamic parameters BP: blood pressure; mmHg: millimeters of mercury; SD: standard deviation; min: minute

Variable	Group A (n = 30)	Group B (n = 30)	p-value
Systolic BP (mmHg), mean ± SD	131.0 ± 15.40	127.67 ± 13.05	0.369
Diastolic BP (mmHg), mean ± SD	80.67 ± 7.40	82.30 ± 7.29	0.393
Heart rate (beats/min), mean ± SD	80.83 ± 9.81	80.83 ± 10.50	1.000
SpO₂ (%), mean ± SD	99.6 ± 0.72	99.5 ± 0.63	0.570

Postoperative pain scores

Postoperative pain scores differed significantly between groups at selected time points. Group A had significantly lower VAS scores immediately after surgery, indicating better very early analgesia (p < 0.001). However, Group B showed significantly lower pain scores at six hours (p < 0.001) and 12 hours (p = 0.028), suggesting more sustained postoperative analgesia. VAS scores were comparable at baseline (p = 0.427) and at 24 hours (p = 0.134) (Table 3).

**Table 3:** Baseline and Postoperative VAS pain scores Data are presented as median(interquartile range) *p < 0.05 is considered significant VAS: visual analog scale ^$^Mann Whitney test

Time point of assessing VAS	Group A (n = 30)	Group B (n = 30)	p-value^$^
Baseline	3(2)	4(1.75)	0.427
0 hour postoperatively	1(0)	2(1)	<0.001*
6 hour postoperatively	4(1.75)	2(1)	<0.001*
12 hour postoperatively	4(0)	4(1)	0.028*
24 hour postoperatively	4(0)	4(0)	0.134

Time to first rescue analgesia

Time to first rescue analgesia was significantly longer in Group B than in Group A. The median time to rescue analgesia was nine hours in Group B, compared with five hours in Group A (p < 0.001), indicating a longer duration of analgesic effect with the bilateral ESP block (Table 4).

**Table 4:** Time to first rescue analgesia Data are expressed as median(interquartile range) *p < 0.05 is considered significant ^$^Mann Whitney test

Variable	Group A (n = 30)	Group B (n = 30)	p-value^$^
Time to rescue analgesia (hours)	5(2)	9(2)	<0.001^*^

Patient comfort and postoperative complications

Patient comfort during the procedure was significantly better in Group B than in Group A. All patients in Group B, 30 (100%), were comfortable, whereas 10 (33.3%) patients in Group A reported discomfort (p < 0.001). Post-procedural backache was uncommon and did not differ significantly between groups (p = 0.313). In contrast, post-procedural hypotension occurred significantly more often in Group A, affecting 10 (33.3%) patients, while no patient in Group B (zero) developed hypotension (p < 0.001) (Table 5).

**Table 5:** Patient comfort and postoperative complications *p < 0.05 is considered significant ^$^Chi-square test

Variable	Group A (n = 30)	Group B (n = 30)	p-value^$^
Comfortable, n (%)	20 (66.7)	30 (100.0)	<0.001^*^
Uncomfortable, n (%)	10 (33.3)	0 (0.0)	
Backache present, n (%)	1 (3.3)	0 (0.0)	0.313
Backache absent, n (%)	29 (96.7)	30 (100.0)	
Hypotension present, n (%)	10 (33.3)	0 (0.0)	<0.001^*^
Hypotension absent, n (%)	20 (66.7)	30 (100.0)	

## Discussion

This randomized double-blind study demonstrated that bilateral ESP block provided more sustained postoperative analgesia than single-shot TEA in patients undergoing laparoscopic abdominal surgery. Although TEA was associated with lower pain scores in the immediate postoperative period, this effect was not sustained. In contrast, the ESP block resulted in lower pain scores at later postoperative time points, a longer time to first rescue analgesia, better patient comfort and no episodes of hypotension or backache.

TEA provides dense segmental blockade of both somatic and visceral afferent fibers, which likely explains the significant early analgesic effect observed in our study, similar to the findings reported by Tzimas et al. in abdominal surgeries [3]. However, as noted by Macpherson et al., the analgesic duration of a single-shot TEA is inherently limited, and the associated sympathetic blockade may result in hypotension, as observed in one-third of patients in our cohort. Although continuous epidural analgesia using an indwelling catheter is the conventional approach for prolonged postoperative pain control, a single-shot TEA was intentionally used in the present study to enable a standardized comparison with the single-injection bilateral ESP block. This hemodynamic effect remains an important limitation of epidural techniques in perioperative pain management [11].

In contrast, the more prolonged analgesic effect observed with ESP block may be attributed to the spread of local anesthetic deep to the erector spinae muscle, with cranio-caudal extension across multiple dermatomes and possible anterior spread to the paravertebral and epidural spaces, as originally described by Forero et al. [4]. Kot et al. and Krishnan and Cascella further noted that ESP block can provide both somatic and visceral analgesia while avoiding the marked sympathetic blockade associated with epidural techniques [5,8]. This mechanism may explain the lower pain scores at six and 12 hours and the significantly longer time to first rescue analgesia observed in our ESP group.

Hemodynamic stability is an important clinical advantage of the ESP block. As it generally does not cause extensive bilateral sympathetic blockade, the risk of hypotension is lower, making it particularly suitable for patients with reduced physiological reserve and for enhanced recovery protocols. In the present study, the complete absence of hypotension in the ESP group reinforces this clinical advantage.

Zubair et al. reported that continuous ESP block provided postoperative analgesia comparable to continuous TEA after living donor hepatectomy, with better hemodynamic stability [1]. Likewise, Kukreja et al. reported that in thoracic surgery patients, ESP block achieved effective postoperative pain control with fewer adverse effects than TEA [6]. In laparoscopic abdominal surgeries, Jain et al., Fu et al., and Tulgar et al. also found that ESP block was associated with improved postoperative analgesia and a longer duration before rescue analgesia was needed [12-14]. Although these studies involved different surgical populations and continuous techniques, their findings are comparable to the present study and further support the role of ESP block as an effective analgesic technique with favorable cardiovascular stability.

An additional important observation in our study was the greater patient comfort seen with the ESP block. This may be attributed to its simpler ultrasound-guided technique, minimally invasive nature, and lower rate of hemodynamic instability. In our study, postoperative hypotension occurred more frequently in the thoracic epidural group, whereas post-procedural backache was infrequent and comparable between the two groups. All patients received a standardized postoperative analgesic regimen, with intravenous paracetamol administered as the first-line rescue analgesic followed by intramuscular butorphanol when additional pain relief was required. These findings further support the favorable safety profile of the ESP block. Kot et al., in their narrative review [5] and Tsui et al., in their pooled review, also highlighted the favorable safety profile and technical ease of the ESP block [15].

This study has a few limitations. It was a single-center study with a relatively small sample size, and outcomes were assessed only during the first 24 postoperative hours. In addition, the study population included patients undergoing different types of laparoscopic abdominal procedures, which may have varied in surgical complexity, extent of tissue manipulation, and postoperative pain intensity, potentially influencing analgesic requirements. Furthermore, TEA was administered as a single-shot technique rather than as a continuous epidural infusion, which is the conventional approach for prolonged postoperative analgesia. This approach was intentionally chosen to enable a standardized comparison with the single-injection bilateral ESP block, thereby minimizing differences attributable to the mode of analgesic delivery. Future multicenter studies with larger sample sizes, longer follow-up periods, recovery-focused endpoints, and more homogeneous surgical populations, as well as comparisons between continuous TEA and continuous catheter-based ESP block, are warranted to further define the relative efficacy and safety of these techniques in laparoscopic abdominal surgery.

## Conclusions

Bilateral ESP block provided more sustained postoperative analgesia than single-shot TEA in laparoscopic abdominal surgery, with longer time to rescue analgesia, greater patient comfort, and superior hemodynamic stability. Although TEA was associated with lower pain scores immediately after surgery, this benefit was not maintained.

Overall, ESP block appears to be an effective and well-tolerated alternative to TEA for postoperative analgesia, especially in patients where hemodynamic stability is desirable.
